# Functional Characterization of a Novel *KCNJ11* in Frame Mutation-Deletion Associated with Infancy-Onset Diabetes and a Mild Form of Intermediate DEND: A Battle between K_ATP_ Gain of Channel Activity and Loss of Channel Expression

**DOI:** 10.1371/journal.pone.0063758

**Published:** 2013-05-07

**Authors:** Yu-Wen Lin, Anlong Li, Valeria Grasso, Domenica Battaglia, Antonino Crinò, Carlo Colombo, Fabrizio Barbetti, Colin G. Nichols

**Affiliations:** 1 Department of Cell Biology and Physiology, and Center for the Investigation of Membrane Excitability Diseases, Washington University School of Medicine, St. Louis, Missouri, United States of America; 2 Laboratory of Mendelian Diabetes, Bambino Gesù Children's Hospital, Research Institute, at University of Tor Vergata, Rome, Italy; 3 Child Neurology Unit, Department of Pediatrics, Sacro Cuore Catholic University, Rome, Italy; 4 Autoimmune Endocrine Diseases Unit, Endocrinology Department, Bambino Gesù Children's Hospital, Research Institute, Palidoro, Rome, Italy; 5 Department of Internal Medicine, University of Tor Vergata, Rome, Italy; University of Houston, United States of America

## Abstract

ATP-sensitive potassium (K_ATP_) channels are widely distributed in various tissues and cell types where they couple cell metabolism to cell excitability. Gain of channel function (GOF) mutations in the genes encoding Kir6.2 (*KCNJ11*) or the associated regulatory ssulfonylurea receptor 1 subunit (*ABCC8*), cause developmental delay, epilepsy and neonatal diabetes (DEND) due to suppressed cell excitability in pancreatic β-cells and neurons. The objective of this study was to determine the molecular basis of infancy-onset diabetes and a mild form of intermediate DEND, resulting from a novel *KCNJ11* in frame mutation plus deletion. The naturally occurring Kir6.2 mutation plus deletion (Ser225Thr, Pro226_Pro232del) as well as the isolated S225T mutation or isolated del226–232 deletion were coexpressed with SUR1 in COS cells in homozygous or heterozygous states. The protein expression and gating effects of the resulting channels were assessed biochemically and electrophysiologically. For both the deletion and point mutations, simulated heterozygous expression resulted in overall increased conductance in intact cells in basal conditions and rightward shifted ATP dose-response curves in excised patches, due to increased intrinsic open probability. Interestingly, homomeric channels for the combined deletion/mutation, or for the deletion alone, showed dramatically reduced channel expression at the cell membrane, which would underlie a reduced function *in vivo*. These results demonstrate that both the mis-sense mutation and the deleted region in the Kir6.2 subunit are important for control of the intrinsic channel gating and suggest that the clinical presentation could be affected by the competition between loss-of-function by reduced trafficking and enhanced channel gating.

## Introduction

ATP sensitive potassium (K_ATP_) channels are widely distributed in various tissues and cell types where they couple cell metabolism to cell excitability [1], [2]. The gating properties that are critical for the physiological function of K_ATP_ channels are their sensitivity to intracellular nucleotides ATP and ADP, whose concentrations fluctuate as metabolism varies. Both Kir6.x and SUR subunits participate in nucleotide regulation of the channel; ATP inhibits channel activity by binding to the Kir6.x subunits, whereas Mg^2+^-complexed ATP and ADP stimulate channel activity by interacting with SUR subunits [3]. For pancreatic β-cells, increase in glucose concentration drives K_ATP_ channels to close, in response to the increase in [ATP]:[ADP] ratio, resulting in membrane depolarization. The depolarization activates voltage-gated calcium channels leading to a rise of intracellular Ca^2+^ and triggering of insulin secretion [1], [2].

More than 65 gain-of-function (GOF) mutations in K_ATP_ channels genes have now been identified from patients with Neonatal Diabetes Mellitus (NDM) [4], [5], [6]. NDM can result from mutations in *ABCC8* (encoding the SUR1 subunit) or *KCNJ11* (encoding the Kir6.2 subunit). *KCNJ11* mutations are typically dominant, and fall into two major categories. In one, the ATP binding affinity is reduced directly by mutation of residues that form the ATP binding pocket or that interfere with the access of ATP to the binding pocket [7]. Alternatively, ATP sensitivity is decreased allosterically via an increase in the intrinsic open probability of the channels. Mutations in the second category can be located far from the ATP binding pocket itself [8], [9]. These mutations keep channels open, typically by reducing ATP sensitivity, and leading to hyperpolarization of beta-cells with reduced insulin secretion. The severity of the clinical presentation correlates with the magnitude of the shift in ATP sensitivity and ranges from mild in the case of transient NDM (TNDM) to permanent NDM (PNDM), to a syndrome that includes developmental delay and epilepsy (DEND), in addition to NDM [10], as a result of channel over-activity in the central nervous system of patients carrying severe *KCNJ11* GOF mutations [5], [11], [12]. It is now established that sulfonylureas, which specifically act by inhibition of K_ATP_ channels, can provide an optimum treatment for the diabetes in many cases, and in some cases can ameliorate the associated neurological disorders in DEND [13], even in the long term (12).

Recently we reported the phenotype and therapy of a patient who presented with diabetes outside the neonatal period (21 months) and with an episode of epilepsy at 10 years of age [14]. A point mutation (S225T) in combination with a 7 amino acid deletion (del 226–232) was identified in one *KCNJ11* allele. To gain insight to the channel basis of this unusual molecular variant, we have now characterized the functional properties of reconstituted S225T, del226–232 and combined S225T plus del226–232 channels in detail. We show that both the 226–232 deletion and the S225T mutation contribute to significantly increased channel activity, due to a rightward shift of ATP-sensitivity caused by an increase in the intrinsic channel open probability. Interestingly, we also found that homomeric del226–232 or S225T plus del226–232 channels exhibit dramatic reduction in cell surface expression, but co-expression of the del226–232 subunit with wild type (WT) subunits at least partially restores surface density of channels, without restoring ATP sensitivity. Homology modeling suggests that the deleted region is in close contact with an identified binding site for Ankyrin-B, an adaptor protein which has been shown to be associated with the C terminus of Kir6.2 subunits [15], potentially explaining the trafficking problem.

## Materials and Methods

### Genetics and molecular Biology

We cloned mouse Kir6.2 into the pcDNA3.1 vector (Invitrogen, Carlsbad, CA) and the parental plasmid DNA was used to generate Kir6.2 mutations using the QuickChange Site Directed Mutagenesis Kit (Stratagene, La Jolla, CA.) The mutations were confirmed by sequencing. Hamster SUR1 was cloned into the pECE expression vector.

### Expression of K_ATP_ channels in COSm6 cells

COSm6 cells (a subclonal line of COS-7 cells, obtained from Dr. Joe Bryan, Pacific Northwest research Institute, originally from the laboratory of J. L. Goldstein, University of Texas Health Sciences, Dallas) were cultured and transfected with cDNA using FuGENE6 Transfection Reagent (Roche Diagnostics, Indianapolis, IN) as previously described [12] and plated on sterile glass coverslips overnight before patch-clamp experiments.

### ^86^Rb^+^ Efflux Assay

COSm6 cells transfected with GFP, SUR1 and Kir6.2 cDNAs were incubated for 5–12 h in culture medium containing ^86^RbCl (1 μCi/ml) 24 hrs post-transfection. Before measurement of ^86^Rb^+^ efflux, cells were incubated for 5 min at room temperature in Krebs-Ringer solution with metabolic inhibitors (2.5 μg/ml oligomycin and 1 mM 2-deoxy-D-glucose). At selected time points the solution was aspirated from the cells and replaced with fresh solution. At the end of a 40 min period, the cells were lysed with 2% SDS. The ^86^Rb^+^ in the aspirated solution and the cell lysate was counted. The percentage efflux at each time point was calculated as the cumulative counts in the aspirated solution divided by the total counts from the solutions and the cell lysates.

### Electrophysiological methods

Membrane patches were voltage-clamped and currents were measured at a membrane potential of −50 mV (pipette voltage, +50 mV), with inward currents shown as upward deflections. Data were collected using the pClamp 8.2 software suite (Axon Instruments) and Microsoft Excel (Microsoft, Redmond, WA). The bath (intracellular) and pipette (extracellular) solution (K-INT) had the following composition: 140 mM KCl, 10 mM Hepes, 1 mM EGTA, pH 7.4. ATP was added as the dipotassium salt.

### Western blot assay

SUR1 expression was assessed by Western blot of SUR1 that was tagged with a FLAG-epitope (DYKDDDDK) at the N-terminus (fSUR1), as described previously [16], from cells co-transfected with WT or mutant Kir6.2 and fSUR1.

### Data analysis

#### Quantitative analysis of ATP inhibition

Channels in inside-out patches were exposed to varying concentrations of ATP in K-INT solution. Channel activity was normalized to that in the absence of ATP. Dose-response curves were fit by the Hill equation (I_rel_  = 1/(1+{[ATP]/K_1/2, ATP_}^H^); I_rel_  =  current in [ATP]/current in zero ATP; H =  Hill coefficient; K_1/2, ATP_  =  [ATP] causing half-maximal inhibition) to averaged data.

#### Estimation of P_o,zero_-*PIP_2_ method*

PIP_2_ was added to the patch until the current reached a saturating level (*I*_PIP2_). This was assumed to represent a maximum *P*_o,zero_ of ∼0.9 [17]. The fractional change in current was calculated as Fold increase  =  *I*_PIP2_/*I*_initial_ where *I*_initial_ is the initial current. *P*_o,zero_ was then estimated as Po, zero  = 0.9/fold increase.

## Results

We previously described a 12 year-old boy with early onset diabetes and mild neurological features [14]. Direct DNA sequencing revealed a novel spontaneous point mutation S225T combined with deletion of amino-acids 226–232 in *KCNJ11* [14]. *In vitro*, the combined mutation results in a K_ATP_ channel with reduced sensitivity to the inhibitory action of ATP, but normal glyburide sensitivity. Accordingly, glyburide improved diabetes control (HbA1c on insulin:52 mmol/mol/6.9%; on glyburide:36 mmol/mol/5.4%) and also performance on motor coordination tests that were impaired before the switch of therapy [14]. In order to determine the molecular mechanisms by which the mutation/deletion affects channel activity, we have now characterized channel activity and expression level for channels with the point mutation or the deletion alone or combined, in homozygous and heterozygous expression.

### Greatly reduced channel activity in homomeric del226–232 channels and S225T plus del226–232 channels in intact cells and in inside-out patch clamp recordings

**Figure 1:**
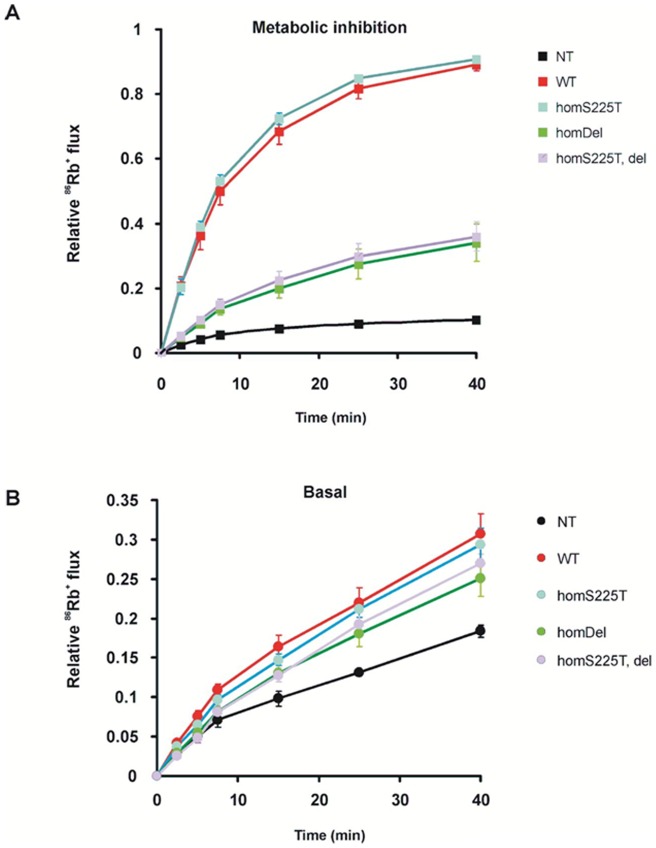
Decreased K_ATP_ activity in both homDel and homT, del channels. Representative ^86^Rb^+^ efflux profile comparing untransfected COSm6 cells (Un) and cells transfected with WT, homS225T, homDel and homT, del channels in metabolic inhibition (A) and in basal conditions (B). Data points indicate means ± SEM of n = 4. NT  =  not transfected.

**Figure 2:**
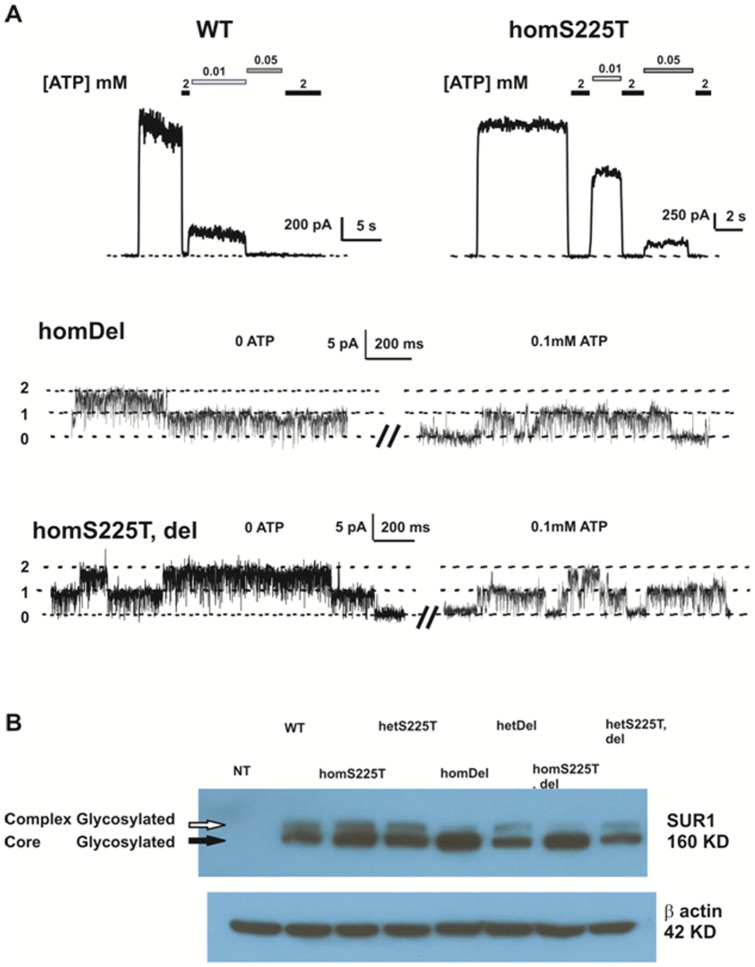
Expression of WT and homomeric mutant channels in patch clamp recordings. (A) representative currents recorded by inside-out excised patch-clamp technique from COSm6 cells expressing WT channels and various mutant channels, at +50 mV pipette potential. Patches were exposed to different concentrations of Mg-free ATP as indicated. Dashed line indicates zero current (WT and homS225T channels) or zero channel level (homDel and homT, del channels). (B) Western Blot of Flag-tagged SUR1 (fSUR1) from various constructs are indicated. The mature (cell surface) complex-glycosylated bands and immature core-glycosylated bands are indicated as upper bands (white arrow) and lower bands (black arrow) respectively. NT = not transfected.

We first assessed K_ATP_ channel activity in intact COS cells by ^86^Rb^+^ efflux assays. Surprisingly, these reveal much lower channel activity induced by metabolic inhibition (MI) in cells expressing homomeric deletion channels (referred to as homDel) or S225T plus deletion channels (referred to as homS225T, del) than in cells expressing WT channels (Fig. 1A). In addition, homDel and homS225T, del channels also display lower ^86^Rb^+^ efflux when compared to WT channels (Fig. 1B). On the other hand, homomeric S225T channels (referred to as homS225T) are slightly more active than WT channels in MI and basal conditions (Fig. 1A and 1B). Taken together, the data suggest that the homDel and homS225T, del channels result in loss of channel function. To further assess channel activity, we measured mutant/deletion channel activity from excised inside-out patch clamp recordings. Representative recordings of channel activities from WT, homS225T, homDel and homS225T, del are shown in Figure 2A. Both WT and homS225T channels display robust currents while only 20% of the patches from homDel and homS225T, del channels displayed even single channel levels of current, and 80% of the patches had no detectable currents (Fig. 2A) (n = 16–19), consistent with the Rb efflux data (Fig. 1). We therefore proceeded to assessment of protein expression level by Western blots. For this, cells were cotransfected with WT or mutant Kir6.2 cDNA and cDNA encoding N-terminally FLAG epitope-tagged SUR1 (referred to as fSUR1), which permits detection of fully assembled channel complexes expressed at the plasma membrane. When co-expressed with either homDel and homS225T, del, fSUR1 showed a significantly reduced complex-glycosylated band in Western blots (Fig. 2B). Reduced complex glycosylation reflects a reduced fraction of the protein that has exited the endoplasmic reticulum and moved past the medial Golgi wherein modification of *N*-linked glycosylation occurs. These results indicate that channels with deletion of these 7 amino acids (homDel and homS225T, del) likely have surface expression defects. On the other hand, the homS225T and WT channels exhibit a similar mature fSUR1 band suggesting that surface expression is unaffected by the point mutation. Interestingly, when the deletion channel was co-expressed with WT cDNA in 1∶1 ratio, the relative density of the mature fSUR1 band was markedly increased compared to the homozygous del expression, indicating rescue by the presence of WT Kir6.2 (see Discussion).

### Deletion of amino acids 226–232 underlies increased K_ATP_ channel activity in simulated heterozygous conditions

**Figure 3:**
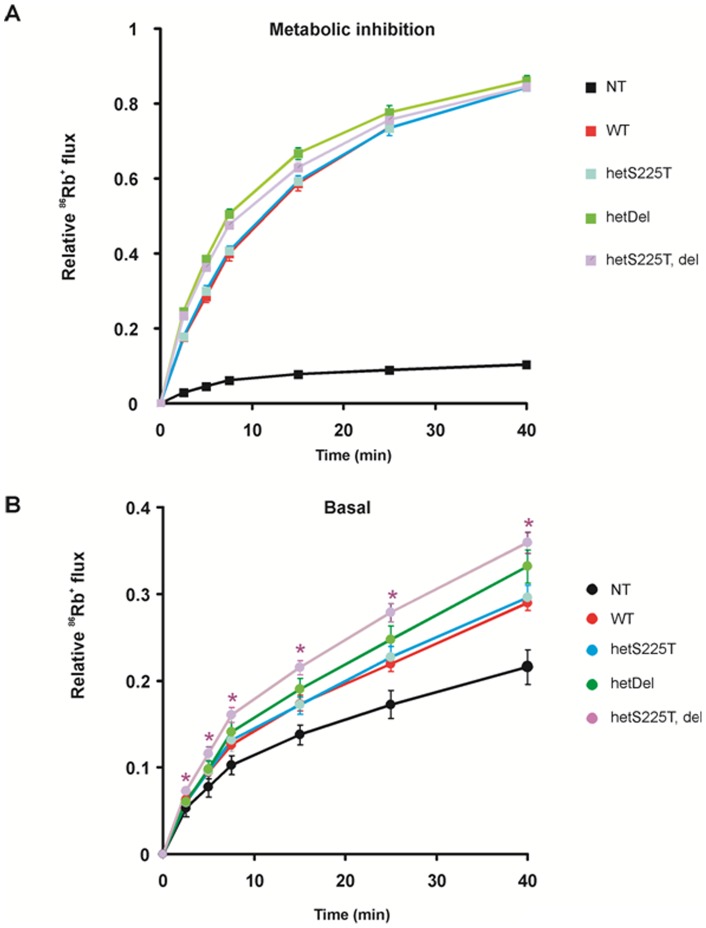
Increased basal K_ATP_ activity in both hetDel and hetT, del channels. ^86^Rb^+^ efflux of mutant Kir6.2 subunits coexpressed with WT subunits in 1∶1 DNA ratio, under metabolic inhibition (A) and in basal states (B). Data points indicate means ± SEM of n = 5. * indicates P<0.05 compared with WT by One-Way ANOVA analysis. NT  =  not transfected (no statistic given).

The loss of surface expression of homDel or homS225T, del channels prohibited further detailed analysis of homomeric channels. We coexpressed the mutant/deletion subunits with WT Kir6.2 in equal ratio (plus SUR1) to simulate the heterozygous state that wil be present *in vivo*, and measured ^86^Rb^+^ efflux across the membrane: heteromeric S225T channels (S225T plus WT in 1∶1 DNA ratio and referred as hetS225T); heteromeric del226–232 channels (referred to as hetDel); heteromeric S225T plus del226–232 channels (referred as hetS225T, Del). In Figure 3A, all four channel types exhibit similar maximal Rb efflux in metabolic inhibition. HetS225T channels exhibit a slight, insignificant, increase in basal flux, but hetDel channels and hetS225T, Del channels both show significantly increased Rb efflux in the basal state, reflecting channel overactivity (Figure 3B).

### Both the deletion and the S225T mutation contribute to the rightward shift of the [ATP]-response curve

**Figure 4:**
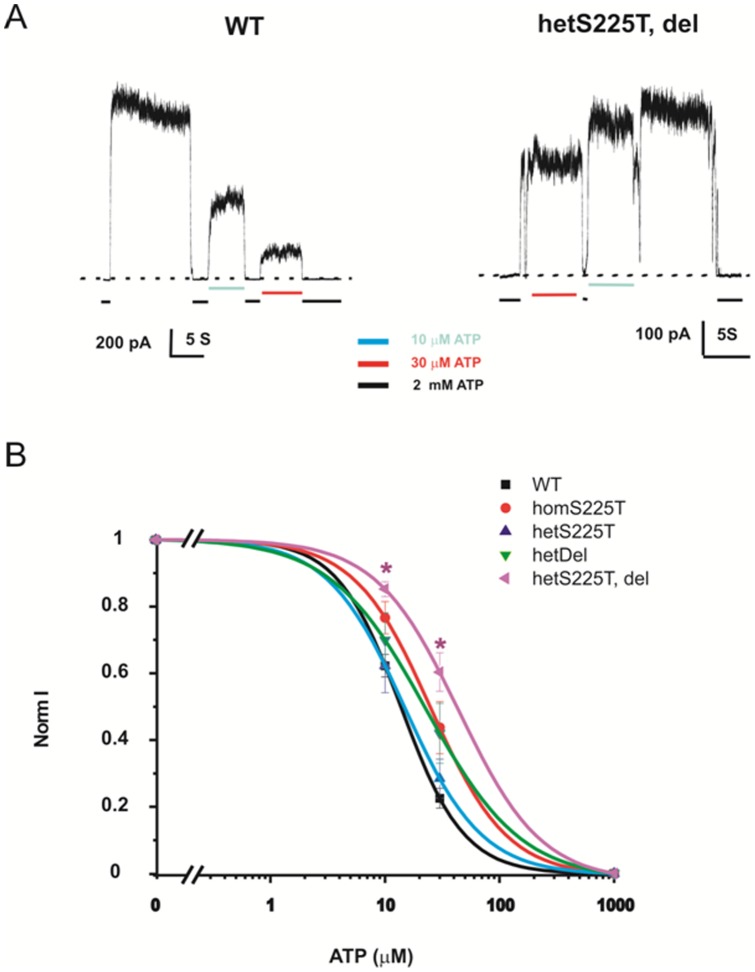
Decreased ATP sensitivity in both hetDel and hetT, del channels. (A) Representative currents recorded by inside-out excised patch-clamp technique from COSm6 cells expressing WT channels and hetT, del mutants. Patches were exposed to different concentrations of Mg-free ATP as indicated. (B) ATP dose-response relationships, fit by Hill equation as described in methods. Data points indicate means ± SEM of n = 5–7 patches. * indicates P<0.05 compared with WT by One-Way ANOVA analysis. The fitted K_1/2_ for WT, homS225T, hetS225T, hetDel, and hetT, del channels are 13.75, 25.06, 14.8, 22.87 and 43.94 (in μM) and the Hill coefficients are 1.6, 1.3, 1.1, 1.28 and 1.2, respectively.

To characterize the mechanisms by which mutant/deletion subunits cause overall gain of channel function in the intact cell, we tested the ATP sensitivity of WT and mutant channels in either homozygous or heterozygous states. Representative recordings of WT and hetT, del channels in response to ATP (in the absence of Mg^2+^) are shown in Figure 4A. A summary of the [ATP]-response curves for WT and mutant channels is shown in Figure 4B. Both hetDel and homS225T channels exhibit slightly right-shifted dose-responses, with a further significantly right-shifted ATP sensitivity of hetS225T, del channels (Fig. 4B) suggesting that both the deletion and the mutation contribute to reduced ATP sensitivity.

### Homology Modeling of Kir6.2 reveals the location of the 225–232 region between two neighboring subunits

**Figure 5:**
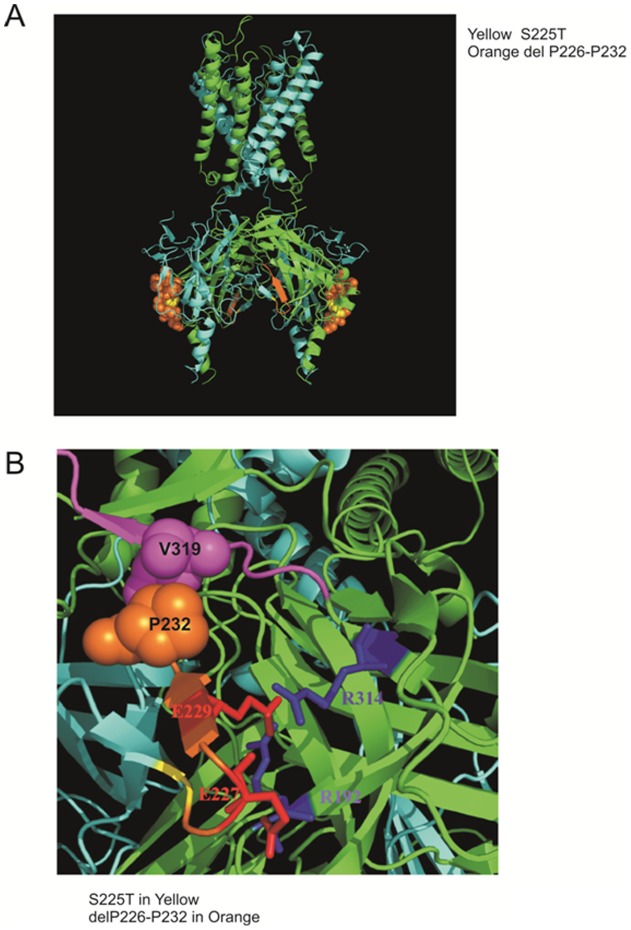
Homology modeling of Kir6.2 from Kir2.2 structure with PyMOL software. (A) S225T is colored in yellow and deleted amino acids 226–232 (-PEGEVVP-) are colored in orange. (B) E227 and E229 are colored and labeled in red while R192 and R314 are colored and labeled in blue. The structure model reveals the possible interaction between the deleted amino acid P232 (orange spheres) and V319 in the proposed Kir6.2 Ankyrin-B binding site (a.a. 316 to a.a. 323 –VPIVAEED- colored in magenta).

**Figure 6:**
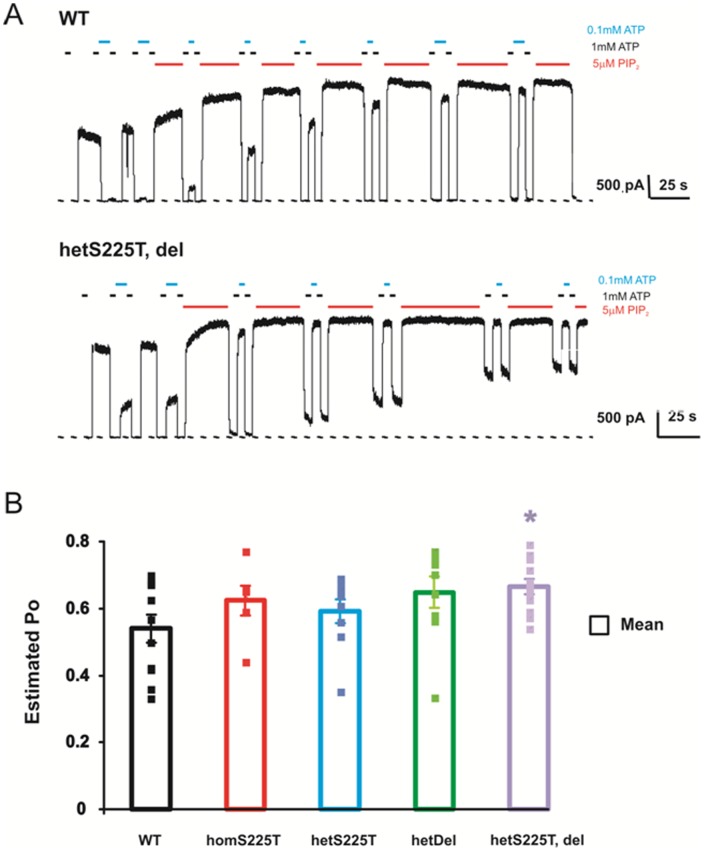
Heterozygous S225T, deletion channels display higher channel open probability, assessed by the ‘PIP_2_’ method. (A) Representative currents recorded by inside-out excised patch-clamp technique from COSm6 cells expressing WT channels and hetT, del mutants. Patches were exposed to different concentrations of Mg-free ATP and PIP_2_ as indicated. (B) Mean estimated Po for various channels: WT (0.53±0.04); homS225T (0.62±0.04); hetS225T (0.59±0.03); hetDel (0.64±0.04); hetT, del (0.66+0.02). * indicates statistically significant difference compared with WT (Student's t-test, p-value <0.05). n = 6–12.

To explore the potential structural basis of the mutagenic effects, we have examined the location of these residues by homology modeling of Kir6.2, based on the Kir2.2 structure [18]. (Figure 5A) This modeling makes clear that S225 and the S226–232 region are located far from the ATP binding pocket, and it is therefore unlikely that either the point mutation or the deletion directly affects ATP binding. Within the deleted region (Figure 5B), residues E227 and E229 have been reported to form inter-subunit ion pairs and thereby affect the intrinsic open probability of the channel [14], [19], such that the open probability of mutations E227K and E229K is greater than that of WT channels [19]. Deletion of these residues might also increase intrinsic open probability and we therefore estimated the open probably for these channels, using the ‘PIP_2_ method’ [17], assessing the increase of channel activity achieved by exposure of excised patches to saturating exogenous PIP_2_. Figure 6A displays representative recordings of WT and hetS225T, del channel activity before and after PIP_2_ application. Open probability (Po) is then calculated as:

Po = 0.9/(fold increase in current in PIP_2_),

where fold increase  =  I _PIP2_/I _initial_. The summary Po values are shown in Figure 6B. Both the deletion and the S225T mutation each contribute to a slightly higher Po in hetS225T, del channels, compared to WT channels, explaining the lower ATP sensitivity (Figure 4) and higher basal Rb efflux (Figure 3) in hetS225T, del channels.

## Discussion and Conclusions

### Gain of channel function due to S225T mutation and deletion of amino acids P226 to P232

Analysis of the component deletion and point mutation reveals a complex contribution of each to the resultant channel phenotype. HomS225T channels display decreased ATP sensitivity (Fig. 2A, 4B) and increased intrinsic Po (Fig. 6B). When expressed together with WT channels, the gain of channel function effect is reduced and there is no significant difference from WT channels in either ATP sensitivity (Fig. 4B) or Po (Fig. 6B). On the other hand, homDel and homS225T, del result in opposing effects of greatly reduced channel activity (Fig. 1 and Fig. 2) in combination with reduced ATP sensitivity (Fig. 2A). However, when coexpressed with WT channels, it is clear that the latter effect dominates, such that heteromeric channels display gain of channel function reflected in increased basal K_ATP_ channel activity in the intact cell (Fig. 3) and decreased ATP sensitivity in excised patches (Fig. 4). Thus, *in vivo*, both the S225T mutation and the deletion will contribute to overactivity as a result of reduced ATP sensitivity, but the net effect on channel activity will be modulated by the reduction of mature channel expression resulting from the deletion (see below).

### Structural basis of increased channel activity

Two heterozygous mutations, E227K and E229K, located within the deletion region, have previously been studied in detail: in heterozygous expression with WT subunits, both generate channels with a significant right shift in their ATP-response curves [19]. Homozygous E227K and E229K channels also display a higher Po in single channels recordings, consistent with our patch clamp data showing that homDel and homS225T, del channels show higher Po and reduced ATP sensitivity (Fig. 2A). Interestingly, other mutations (E227A and E229A) at these same residues have been shown to cause rapid current decay (inactivation) due to the loss of inter-subunit interactions [14]. Based on these studies, E229 was proposed to form an ion pair with R314 from the adjacent subunit, such that disruption of this interaction would lead to inactivation. As revealed by the homology modeling in Figure 5B, E227 might also interact electrostatically with R192 [14]. Although no molecular mechanism so far has been proposed to explain the higher Po in E227K and E229K mutations, conceivably this may relate to repulsive interactions with R314 or R192.

### Implications for unusual presentation of diabetes with epilepsy

The patient carrying the S225T, del mutation had infancy-onset diabetes, as well as learning difficulties during primary school, and a single episode of seizures at 10 years of age [14]. Thus the patient could be categorized as exhibiting an intermediate DEND phenotype [4], and consistent with the gain-of-function phenotype exhibited by the mutant channels. However, the patient first presented with diabetes at 21 months of age, a relatively late presentation when compared to classical neonatal diabetes (i.e. within six months of birth [4]), and not consistent with a mutation severe enough to cause the neurological phenotype. As we show, the deletion causes a dramatic loss-of-function in homozygous expression in COS cells (Fig. 2). The model structure (Fig. 5B) reveals the possible interaction between the deletion amino acid P232 with V319 which is located in the proposed Kir6.2 Ankyrin-B binding site [15]. Ankyrin-B has been shown to regulate the expression and membrane targeting of Kir6.2 in addition to modulating K_ATP_ channel ATP sensitivity [15]. Understanding of the control of K_ATP_ subunit trafficking remains rudimentary but, conceivably, deletion of the Ankyrin-B binding site could result in decreased membrane expression and reduced K_ATP_ currents [20] in a tissue-specific pattern such that the trafficking defect is more dominant in the pancreas, such that the net GOF phenotype is less severe in this tissue, explaining the late presentation of diabetes.
